# Concentrations and sources of polycyclic aromatic hydrocarbons in surface coastal sediments of the northern Gulf of Mexico

**DOI:** 10.1186/1467-4866-15-2

**Published:** 2014-03-19

**Authors:** Zucheng Wang, Zhanfei Liu, Kehui Xu, Lawrence M Mayer, Zulin Zhang, Alexander S Kolker, Wei Wu

**Affiliations:** 1 Department of Geography, Northeast Normal University, Changchun, China; 2 Marine Science Institute, The University of Texas at Austin, Port Aransas, TX, USA; 3 Department of Oceanography and Coastal Sciences, Louisiana State University, Baton Rouge, LA, USA; 4 Coastal Studies Institute, Louisiana State University, Baton Rouge, LA, USA; 5 School of Marine Sciences, University of Maine, Walpole, ME 04573, USA; 6 The James Hutton Institute, Aberdeen, UK; 7 Louisiana Universities Marine Consortium, Chauvin, LA 70344, USA; 8 Department of Coastal Sciences, Gulf Coast Research Laboratory, The University of Southern Mississippi, Ocean Springs, MS 39564, USA

**Keywords:** Polycyclic aromatic hydrocarbons, Grain size, Surface area, Organic carbon, Principal component analysis, Bioavailability, Coastal sediments, Northern Gulf of Mexico

## Abstract

**Background:**

Coastal sediments in the northern Gulf of Mexico have a high potential of being contaminated by petroleum hydrocarbons, such as polycyclic aromatic hydrocarbons (PAHs), due to extensive petroleum exploration and transportation activities. In this study we evaluated the spatial distribution and contamination sources of PAHs, as well as the bioavailable fraction in the bulk PAH pool, in surface marsh and shelf sediments (top 5 cm) of the northern Gulf of Mexico.

**Results:**

PAH concentrations in this region ranged from 100 to 856 ng g^−1^, with the highest concentrations in Mississippi River mouth sediments followed by marsh sediments and then the lowest concentrations in shelf sediments. The PAH concentrations correlated positively with atomic C/N ratios of sedimentary organic matter (OM), suggesting that terrestrial OM preferentially sorbs PAHs relative to marine OM. PAHs with 2 rings were more abundant than those with 5–6 rings in continental shelf sediments, while the opposite was found in marsh sediments. This distribution pattern suggests different contamination sources between shelf and marsh sediments. Based on diagnostic ratios of PAH isomers and principal component analysis, shelf sediment PAHs were petrogenic and those from marsh sediments were pyrogenic. The proportions of bioavailable PAHs in total PAHs were low, ranging from 0.02% to 0.06%, with higher fractions found in marsh than shelf sediments.

**Conclusion:**

PAH distribution and composition differences between marsh and shelf sediments were influenced by grain size, contamination sources, and the types of organic matter associated with PAHs. Concentrations of PAHs in the study area were below effects low-range, suggesting a low risk to organisms and limited transfer of PAHs into food web. From the source analysis, PAHs in shelf sediments mainly originated from direct petroleum contamination, while those in marsh sediments were from combustion of fossil fuels.

## Introduction

As a major group of persistent organic pollutants, polycyclic aromatic hydrocarbons (PAHs) are widely found in natural environments. The geochemical behaviors of PAHs have been widely studied because of their carcinogenic, mutagenic and persistent properties [1-6]. Determining PAH concentrations in coastal and oceanic sediments is necessary for risk assessment and evaluation of ecosystem health [3,7,8]. PAHs in the environment are petrogenic and pyrogenic, which affects their composition and bioavailability. For example, PAHs sourced from oil are more bioavailable than those from coal [9]. In coastal environments most PAHs derive from petroleum spillage, industrial discharges, atmospheric deposition, and urban run-off [10]. PAHs in the environment may be sorbed onto particles and deposited into sediments due to their highly hydrophobic nature [11-13]. The strong adsorption of PAHs to sediment particles may lead to their low bioavailability and biodegradation rate, preserving them in sediments for an extended time period.

Concentrations of PAHs in sediments are controlled by organic matter content and grain size [14-16]. Organic matter plays a major role in sorbing PAHs, particularly when its content in sediments is >0.1% [17]. The types of organic matter may also affect PAH concentrations in sediments [18]. For example, condensed carbonaceous geosorbents, such as black carbon, coal and char, have greater sorption capabilities compared to natural amorphous organic matter [19]. Humic substances, geopolymers and materials from combustion (soot or black carbon) differ in their capacity for sorption/desorption of PAHs [20]. The role of grain size in PAH sorption is still under debate. Rockne et al. [21] found high PAH concentrations in large size fractions (>500 μm), while other studies suggested that high PAH concentrations associate with smaller size fractions [22]. However, Yang et al. [23] suggested that PAHs associate with the low density fraction dominated by plants- and coal-derived debris rather than particle size. Mostafa et al. [24] suggested that the distribution and concentration of PAHs in sediments are determined by their contamination sources rather than the type of sediment.

The northern Gulf of Mexico is a major hub of oil and gas industries in the United States. The offshore areas produce about 1.3 million barrels of crude oil per day, which amounts to ~23% of the total US production, while the onshore areas account for 40% of total petroleum refining capacity (http://www.eia.gov). In addition, this region contains abundant gas hydrate deposits and oil seeps, which have a high potential to release organic contaminants into sediments [25,26]. Considering that PAHs account for 10-45% of total hydrocarbons in crude oil [11,27], and the importance of this region to US fisheries stock and migratory waterfowl [28], it is important to understand the distribution of PAHs in this area. Previous studies showed that high PAH concentrations in shallow Gulf of Mexico sediments (<20 m) were found in the early 1970s, when gas and oil production was highest [25,26,29]. Wade et al. [30] determined concentrations of trace metals and PAHs in deep Gulf of Mexico sediments, and suggested that PAH levels are associated with drilling operations. However, few studies have compared the PAHs distribution in shallow shelf and marsh sediments from the Gulf of Mexico. In particular, contamination sources and association mechanisms of PAHs in these sediments remain unclear. The goals of this study were to: (1) determine the concentration and composition of PAHs, (2) identify contamination sources of PAHs, and (3) elaborate on the factors controlling distribution, composition and bioavailability of PAHs in surface marsh and shelf sediments in the northern Gulf of Mexico.

## Materials and methods

### Sample collection

**Figure 1:**
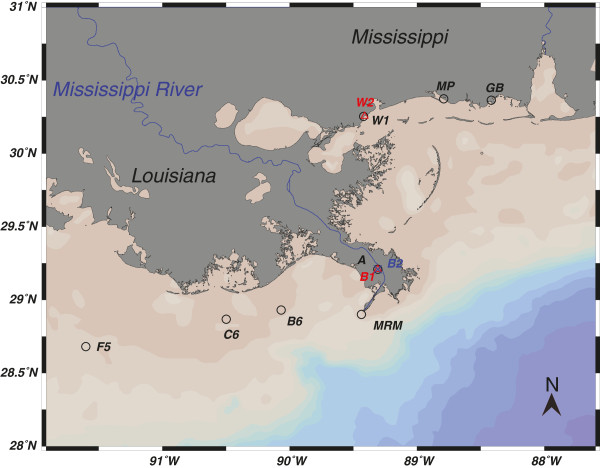
**Sampling stations in the northern Gulf of Mexico.** Stas. C6, CT, F5 and B6 were from marine shelf; Stas. Waveland H, W1, GB, and MP were from salt marsh; MRM from the Mississippi River mouth; A, B1, B2 were from estuarine freshwater marsh.

Shelf sediments were collected in the Mississippi River plume off the Louisiana coast (<30 m water depth) using a HYPOX corer [31] in May 2010 (Figure 1). The sampling stations on the shelf were named after the long-term hypoxia study in this area [32,33], including C6, CT, F5, B6, and MRM. Sediments at Sta. MRM (Mississippi River mouth) were also collected in August 2010 and May 2012. Marsh sediments along the adjacent coast were collected in August 2012 using a home-made corer [34], including salt marshes Waveland 1 (W1), Waveland 2 (W2), Marsh Point (MP) and Grand Bay (GB), and freshwater marshes (A, B1, B2). Surface sediments (0–5 cm) were sectioned and stored in pre-combusted glass jars in a freezer (−20°C) until analysis. Before analysis, sediments were freeze-dried and ground to achieve homogeneity.

### Sediment properties

Total organic carbon (TOC) and total nitrogen (TN) in sediment were measured by a CHN elemental analyzer, after the carbonates were removed by acid fuming in a sealed container with concentrated HCl [35]. The grain size of surface sediments was measured using a Beckman-Coulter laser particle size analyzer (Model LS 13 320). Briefly, 20 mL deionized water was added to 1 g of freeze-dried sediment in a beaker. After soaking for 24 h, the sediment was subjected to vortex mixing for 5 min to disaggregate loosely-attached aggregates. Neither organic matter nor carbonate was removed for the laser grain size analysis. The size range of detection for this analyzer is from 0.02 to 2000 μm. Mineral specific surface area was measured after the sediment was muffled overnight at 350°C, using the one-point BET method on a Quantachrome Monosorb analyzer [36].

### PAHs extraction from sediments

Sixteen priority PAHs listed by the US EPA were analyzed, including naphthalene (Nap), acenaphthene (Ace), acenaphthylene (Acy), fluorene (Fl), phenanthrene (Phe), anthracene (An), fluoranthene (Flua), pyrene (Pyr), benzo[a]anthracene (BaA), chrysene (Chr), benzo[b]fluoranthene (BbF), benzo[k,j]fluoranthene (BkF), benzo[a]pyrene (BaP), indeno[1-3]pyrene (InP), dibenzo[a,h]anthracene (DBA), and benzo[ghi]perylene (BgP). A mixture of targeted standards for calculation and three deuterated PAHs as the surrogate standards, D_10_- acenaphthene (Ace-*d*_10_), D_10_- phenanthrene (Phe-*d*_10_), and D_12_- benzo[a]pyrene(BaP-*d*_12_) (Sigma), were used in this study.

The PAH extraction from sediment followed the protocol of Rhind et al. [37]. Briefly, ca. 1 g sediment (dry weight) was added with surrogate standards (Ace-d10, Phe-d10, BaP-d12) and 8 mL ethanoic potassium hydroxide (1 M). The samples were heated to 90°C for 8 h. The analytes were extracted by hexane and then purified with a column packed with activated silica gel and topped with 1 cm anhydrous sodium sulfate. The PAHs were then eluted with dichloromethane/hexane (1:4, v/v). The eluted solution was concentrated and exchanged by hexane to 1 mL with a rotary evaporator, and stored at 4°C until further analysis.

### Bioavailable PAH extraction from the sediments

Bioavailable PAHs were extracted from sediments of several stations based on sample availability, including W1, W2, A, B1, MRM, and C6. The extraction protocol followed the study of Voparil and Mayer [38], which mimics the fraction of PAH available to a marine benthic invertebrate such as a polychaete. The animal species to which this protocol was tuned lies at the approximate median of 18 species of deposit-feeder dissolution capability for PAH [39]. Briefly, 10–20 g of sediment was extracted using a cocktail of 15 mM sodium taurocholate and 5 g L^−1^ protein in a Ca-free, artificial seawater matrix. The ratio of sediment to cocktail was 0.25. The slurry was centrifuged at 4000 rpm for 12 min and the supernatant was filtered through pre-combusted GF/F filters. PAHs in the filtered solution were spiked with surrogate standard Phe-*d*_10_ 20 uL (10 ng/uL), and serially extracted with 25 mL DCM for three times. The combined extractions were passed through sodium sulfate, rotovapped, and purified by a self-packed chromatography column (top with ~ 2 cm anhydrous sodium sulfate and bottom with 3 g silica gel), exchanged by hexane to 1 mL with a rotary evaporator, and stored in a refrigerator at 4°C until further analysis.

### PAH analysis

PAHs were analyzed by gas chromatography–mass spectrometry (GC-MS, Shimadzu QP2010 plus). The GC-MS is equipped with a RXi-1MS capillary column (20 m × 0.18 mm i.d., film thickness 0.18 μm), with helium as the carrier gas at a flow rate of 0.8 mL min^−1^, using a selective ion monitoring mode to detect PAHs. The scan ions ranged from 126 to 279 atomic mass units, and the dwell time per ion was 200 milliseconds. The oven temperature was held at 60°C for 1 min, increased to 240°C at a rate of 10°C min^−1^, and then increased to 280°C at a rate of 4°C min^−1^ and held for 3 min. The temperatures of the injector and detector were 260°C and 275°C, respectively. The injection volume was 1 μL with a split ratio of 1/20. All of the 16 PAHs were eluted from 5 to 30 min in the GC column.

### Quality assurance/quality control (QA/QC)

All analyses were conducted on duplicate samples (except shelf sediments), and the size distribution analysis was conducted on triplicate samples. Three deuterated PAHs (Ace-*d*_10_, Phe-*d*_10_, BaP-*d*_12_) were used as surrogates during the extraction for recovery calculation. The average recovery rates of the three surrogates for sediments ranged from 65.2% to143.0% (n = 49). The variance of replicate analyses for bioavailable PAHs was 3% (1 relative SD). The method blank was analyzed by the same procedure as the samples, and the background contamination was negligible.

## Results

### Sediment characterization

TOC and TN contents in the marsh sediment ranged from 0.1 to 6.9%, and 0.02 to 0.71%, respectively, both of which were higher compared to shelf sediments, which ranged from 0.6-1.2% and 0.07-0.11%, respectively (Table 1). Temporal variability or spatial heterogeneity was significant for TOC in MRM sediments, ranging from 1% (May) to 13.9% (August). Atomic C/N ratios of sedimentary OM varied from 7.2 to 19.5, with higher values in marsh than shelf sediments. This pattern is expected because of the dominant marsh plant input to marsh sediments as compared to algal input to shelf sediments.

**Table 1:** Sediment properties for each station (nd: not detected)

**Sampling station**	**TOC (%)**	**TN (%)**	**C/N**	**Sediments fractions (%)**			**Surface area (m**^**2**^**/g)**
				**Sand (>63 μm)**	**Silt (4 ~ 63 μm)**	**Clay (<4 μm)**	
MP	2.02	0.13	15.44	71.46	21.93	6.61	3.87
GB	6.93	0.43	16.12	63.61	31.24	5.15	8.16
W1	0.94	0.05	17.20	65.92	29.17	4.91	4.43
W2	1.35	0.10	13.81	61.09	30.77	8.14	4.87
B6	1.15	0.11	10.10	11.37	70.02	18.61	16.59
C6	0.70	0.07	9.70	21.43	62.88	15.69	13.88
CT	0.71	0.07	10.10	12.40	69.06	18.54	16.05
F5	0.62	0.08	8.00	28.12	59.47	12.41	13.21
A	0.11	0.02	7.24	94.55	4.07	1.38	1.34
B1	0.45	0.04	9.11	92.29	6.20	1.51	0.90
B2	0.98	0.05	18.80	61.22	34.11	4.67	3.53
MRM-2010-05	1.36	0.14	10.10	15.16	66.12	18.72	nd
MRM-2010-08	13.89	0.71	19.50	10.86	67.13	22.01	13.08
MRM-2012-05	0.97	0.10	10.10	10.00	59.88	30.12	nd

TOC and TN represent total organic carbons and total nitrogen, respectively. Silt and clay fractions decreased from shelf sediments to coastal sediments, as well as surface area. The TOC% and C/N exhibited opposite patterns.

The shelf sediments contained >60% silt (4–63 μm), whereas the marsh sediments contained >60% sand (>63 μm). Specific mineral surface area in shelf sediments ranged from 13 to 16 m^2^ g^−1^, typical ranges found in coastal sediments [36]. The specific surface area of shelf sediments was relatively uniform, suggesting the homogeneity of mineral grains due to extended physical dynamics on the shelf. In contrast, surface areas in marsh sediments were lower and were more variable, as expected from the coarser grains, ranging from 0.9 to 8.2 m^2^ g^−1^. The wide range of surface area in the marsh sediment implies high heterogeneity of mineral grains in marsh sediments. For example, silts and clays accounted for 40% at Sta. B2 but <10% at Sta. B1, even though these two stations are only 3.2 km apart.

### Concentrations and compositions of PAHs in sediments

**Figure 2:**
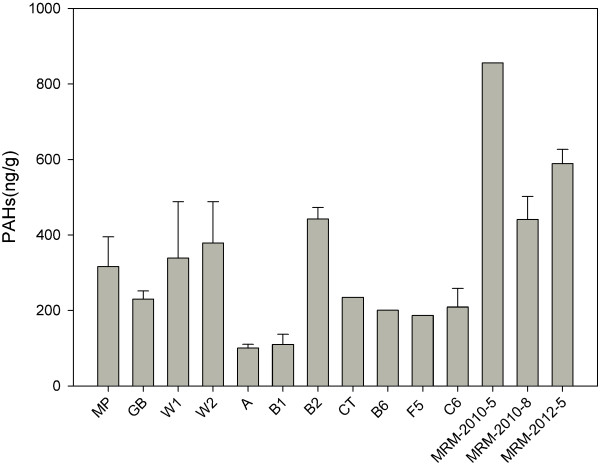
**Total PAH concentrations in sediments from northern Gulf of Mexico.** Error bars represent one standard deviation of duplicate samples.

PAH concentrations were higher in marsh sediments (229–379 ng g^−1^) than shelf sediments (175–244 ng g^−1^). Highest PAH concentrations occurred at Sta. MRM (441–856 ng g^−1^), with concentrations in May 2010 up to 2 times higher than in August 2010 and May 2012 (Figure 2). This large variation indicates an impact of contaminant source and strong physical dynamics at the river mouth. Similarly, PAH concentrations in the Mississippi River marsh sediments exhibited large differences. Stations B1 and B2 are within 3.2 km distance, but their PAH concentrations differed by four fold at Stations B1 and B2 (Figure 2). This difference is likely due in part to the fact that these areas represent newly forming land, which emerged following the Great Mississippi River flood of 2011. Such newly formed land often features a diversity of sediment types and depositional environments located within close proximity to each other [40].

High PAH concentrations in sediments may be expected based on the significant level of petroleum and gas activities in the area. The concentrations measured (229–379 ng g^−1^) were lower than the ERL (effects range-low) value established by Long et al. (1995) [41], suggesting that these PAH levels present little risk to organisms in this area. These concentrations were generally consistent with previous studies from this area [29,30], and much lower than highly industrialized estuaries such as the Lenga (290–6118 ng g^−1^) [42], Patos Lagoon (38–11780 ng g^−1^) [43], and Jiulong River (280–1074 ng g^−1^) [44]. Shelf sediments were collected during the *Deepwater Horizon* oil spill and marsh sediments were collected two years after the spill, so hydrocarbon contamination might be expected. Low PAH concentrations detected in the shelf sediments suggested that they were not contaminated to a detectable degree, consistent with the fact that oil mousses and slicks were observed only at the sea surface [45]. Salt marshes in this region may have been impacted by the oil, but two years of weathering can significantly degrade the characterizable hydrocarbons [46]. At the Waveland marshes, Shoreline Cleanup and Assessment Teams (SCAT) reports indicate these sites received, at their maximum (April 13, 2011 report), little or no oiling (gomex.erma.noaa.gov). In contrast, the Mississippi River mouth stations A, B1 and B2 were not oiled during the *Deepwater Horizon* oil spill, as these areas emerged following a massive flood of 2011, and were not extant during the spill [47]. Overall, we found little impact of the *Deepwater Horizon* oil spill on PAH concentrations in the sediments collected.

**Figure 3:**
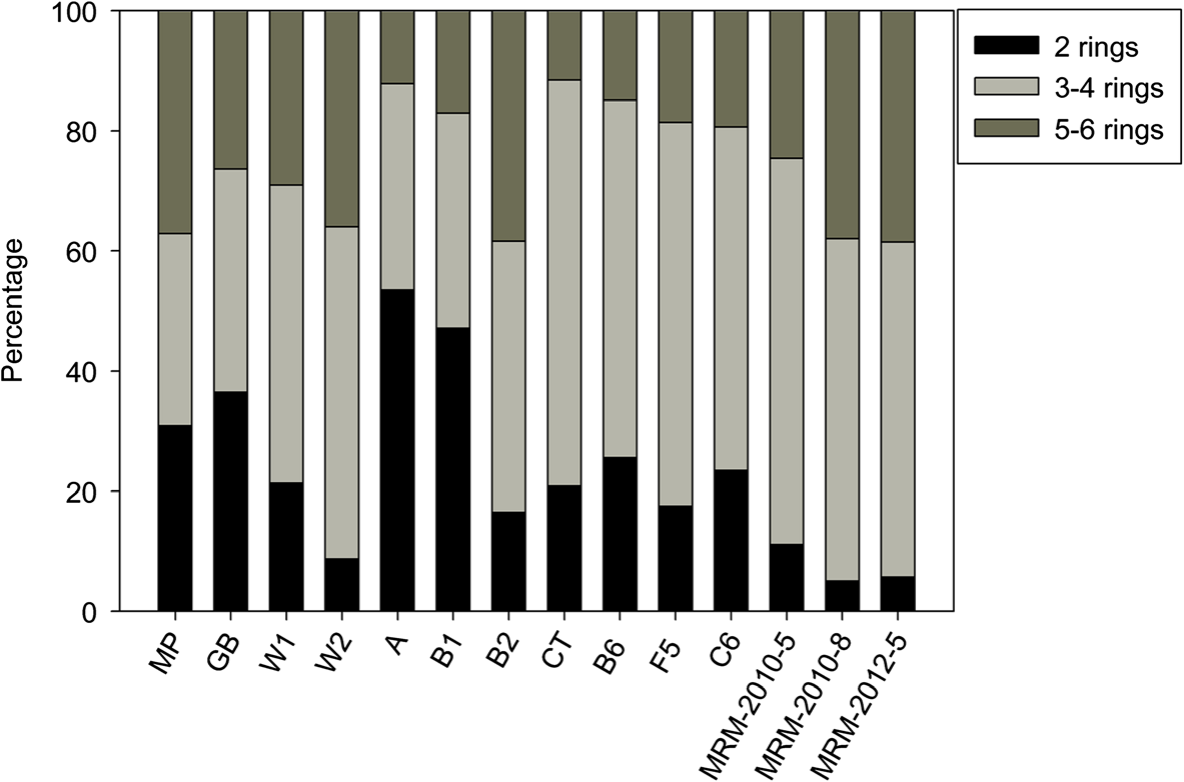
The distribution of PAHs with 2, 3–4 and 5–6 rings in sediments.

Compositionally, PAHs with 3–4 rings accounted for more than 50% of the total PAHs at all stations except Stas. A and B1, where 2-ring PAHs dominated. However, compositions of other PAHs varied among the sampling areas. Proportions of 2-ring PAHs were higher than 5-6-ring PAHs in shelf sediments, opposite to that in marsh sediments (Figure 3). Compositions of PAHs exhibited differences even among the adjacent stations in marsh sediments. For example, Sta. B1 contained a higher proportion of 2-ring PAHs (>40%) and a lower proportion of 5-6-ring PAHs (<20%), while Sta. B2 contained a lower proportion of 2-ring PAHs (<20%) and more 5-6-ring PAHs (>35%).

### Bioavailable PAHs

**Figure 4:**
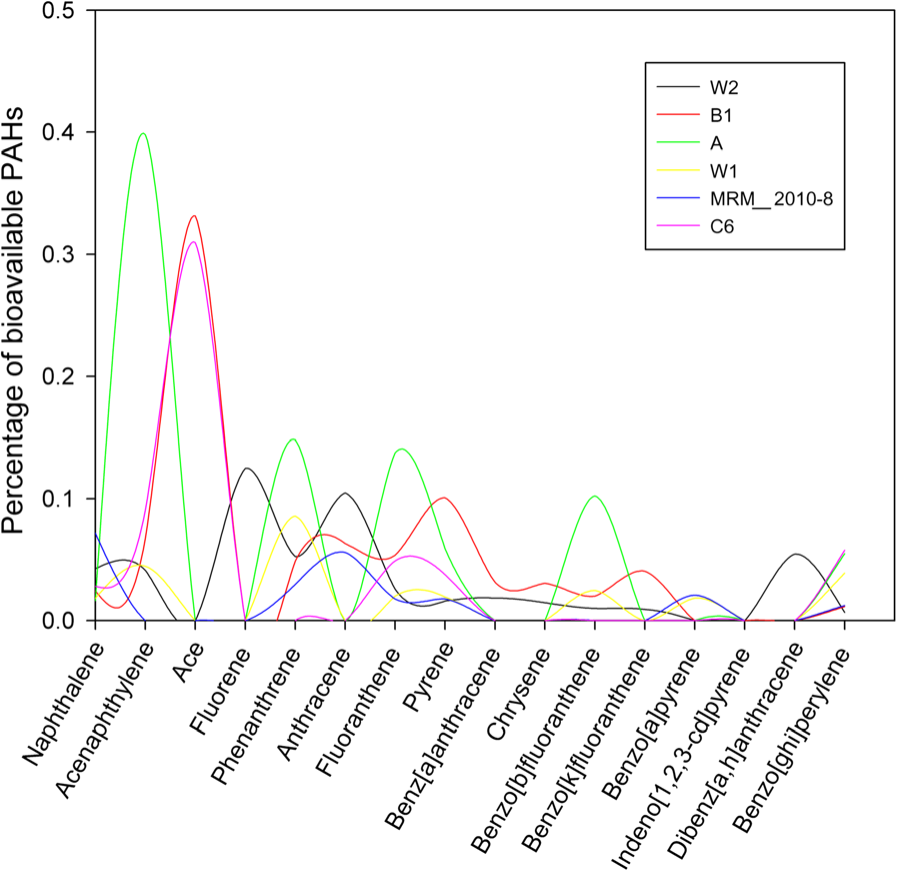
Distribution of bioavailable proportions of PAHs, expressed as percentage of total extractable contents.

The proportions of bioavailable PAHs in total PAHs were low, ranging from 0.02 to 0.06% - a range similar to those found for sediments in other areas [38]. The highest proportion of bioavailable PAHs was found in Sta. A sediment, with low TOC content (0.11%) and small specific surface area (1.2 m^2^ g^−1^). PAHs in Sta. MRM and Sta. C6 sediments exhibited the lowest bioavailable proportions (0.02%). The bioavailable proportions of PAHs in marsh sediments were generally > 0.02%, higher than those in shelf sediments. The types of bioavailable PAHs differed among the sediments. For example, only 7 PAHs were measurably bioavailable in Sta. MRM sediments, as compared to 12 PAHs in Sta. B1 sediments. Although the bioavailable fractions of certain low-molecular-weight PAHs were higher, such as Acy in Sta. W2 sediment (40%) and Ace in Sta. B1 and Sta. C6 sediments (33% and 31%, respectively), bioavailable proportions of most PAHs were <0.05%. The bioavailability of PAHs generally decreased with increasing molecular weight (Figure 4), although the bioavailable proportions of Nap were smaller than those of 3- or 4-ring PAHs at certain stations, such as Stas. A and W2.

## Discussion

### Factors affecting PAH concentrations

**Figure 5:**
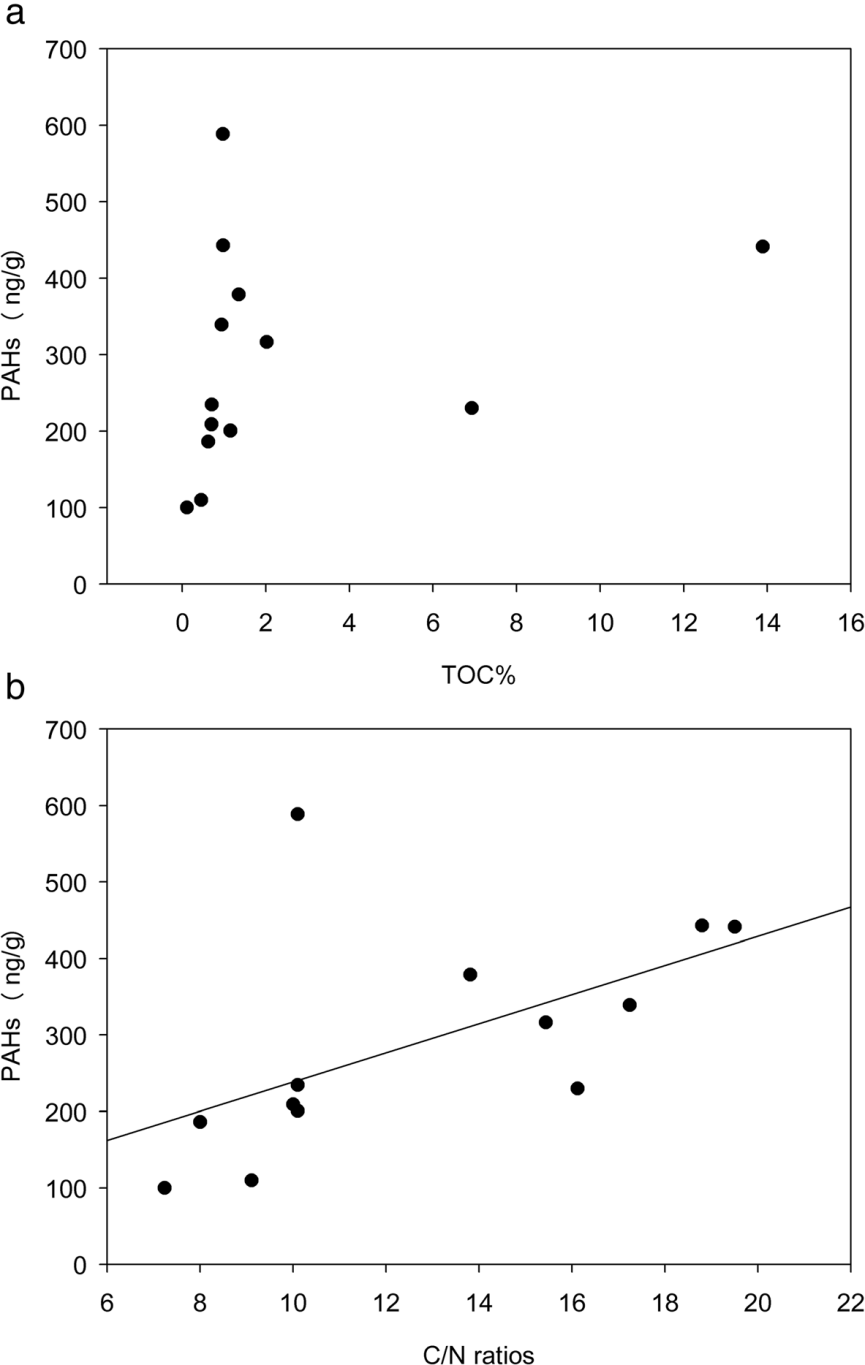
**Relationship between PAH concentrations and (a) TOC (r = 0.3), and (b) C/N ratios (r = 0.75, p < 0.01) in sediments.** The MRM sediment in May 2010 was excluded due to its exceptionally high PAH concentration.

Higher PAH concentrations found in the marsh compared to shelf sediments may provide insight into factors affecting PAH concentrations. Certain sediment properties can affect PAH distribution and concentrations, such as TOC and/or clay content [24]. Specifically, PAH concentrations tend to be higher in sediments with higher TOC contents due to the high sorption capacity of OM [48]. However, a poor correlation was found between TOC and PAH concentrations in this study (Figure 5a, R = 0.30). PAH concentrations were positively correlated with C/N ratios of sedimentary OM (Figure 5b, R = 0.75, p < 0.01). Note that the MRM sediment in May 2010 was not included in this particular analysis because of the exceptionally high PAH concentration, which may be due to upstream effluents. There should be a large amount of contaminants from upstream as the Mississippi River drains the third largest watershed on earth, with much of that land devoted to industry and agriculture [33,49]. C/N ratios can be used to distinguish the source of OM [50]: higher C/N ratios (13.7 ± 4.3) in the marsh sediments indicates a mixture of vascular plant and marine algal sources, whereas the lower C/N ratios in shelf sediments (9.6 ± 0.9) indicates that organic matter was derived more from phytoplankton. The positive relationship between PAH concentrations and C/N ratios suggests that PAHs preferentially attach to terrestrial and marine grass OM than to marine algal OM. Terrestrial OM contains more aromatic moieties than marine OM [51,52], so a tight association of terrestrial OM with the aromatic PAHs was expected. It is also possible that soot particles, which are depleted in nitrogen and are strong sorbents for PAHs [53,54], were more abundant in OM with high C/N ratios. If this hypothesis were true, the proportion of soot particles must be high enough to drive up the C/N ratios of sedimentary OM. However, combusted sediments (BC) and untreated sediments (OC + BC) often have similar C/N ratios for sediments containing 1-4% TOC [55], indicating that soot particles cannot drive C/N ratios significantly due to their small fractions in total OM. Overall, our results suggest that the type of OM, marine vs. terrestrial, is a major factor affecting PAH concentrations in coastal sediments of the northern Gulf of Mexico.

**Figure 6:**
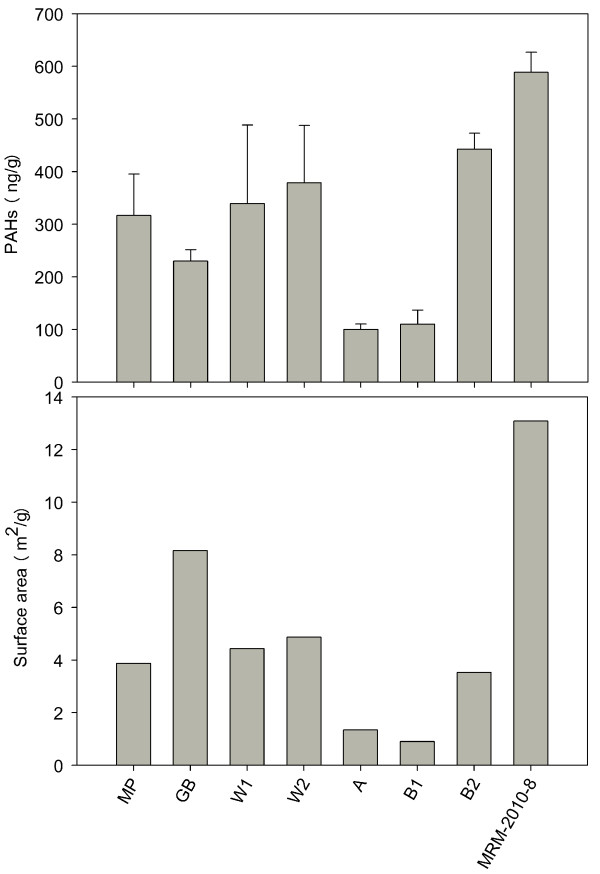
PAH concentrations and specific mineral surface area in marsh sediments.

Mineral surface area affects the distribution of PAHs in sediments, as sorption of PAHs occurs either on mineral surfaces directly or in organic matter coatings on mineral surfaces [56]. Smaller minerals, with high specific surface area, can sorb TOC and organic pollutants, such as PAHs and PCBs [14,15,57,58]. However, PAH concentrations in shelf sediments, with finer minerals (silts and clays) and higher specific surface area, were lower than those in marsh sediments with coarser minerals and lower surface areas (Table 1, Figure 2). This pattern suggests that other factors, such as the type of organic matter or contamination source, are more important in controlling PAH concentrations. This pattern could also be explained by the role of marsh sediments in “biofiltering” PAHs before they are exported to shelf sediments [4], even though a major fraction of sediment may be directly exported from river to shelf environments. Concentrations of PAHs did not correlate with specific surface area over all stations, but they did correlate well if only the marsh sediments are considered (R = 0.70, p < 0.01, Figure 6). This result suggests that when the type of OM and the contamination source are similar (terrestrial OM in this case), mineral surface area may be important in controlling the distribution of PAHs. Consistently, our previous study showed that finer fractions contained higher PAH levels than those coarser fractions after salt marsh sediments from a station in the western Gulf of Mexico were size-fractionated [59].

### Insights from PAH compositions: contamination sources and physical dynamics

**Figure 7:**
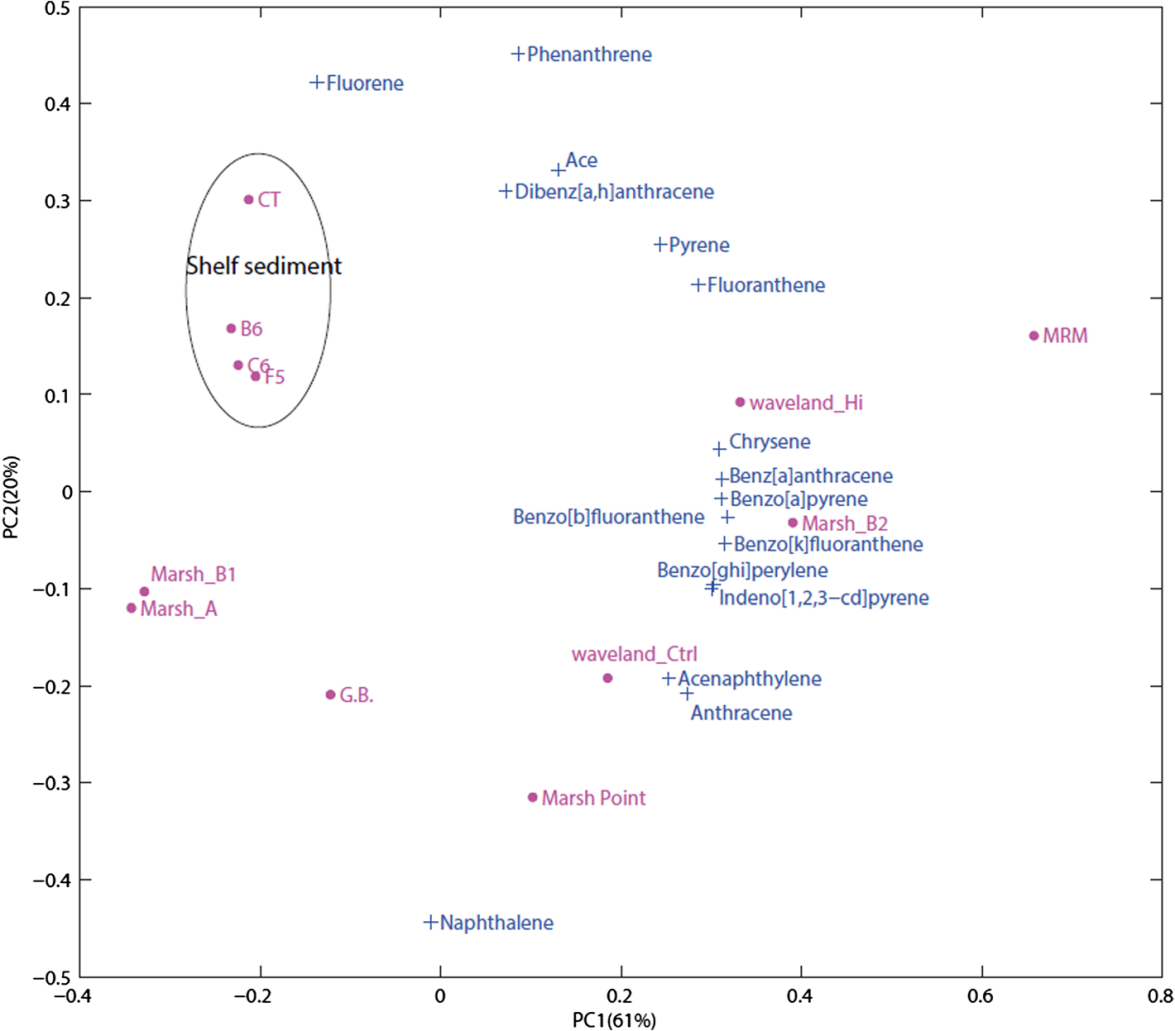
**Principal component analysis (PCA) on PAH composition in sediments.** PC1 explained 61% of the variance of the data matrix, and PC2 20% of the rest. Shelf sediments were clearly separated from marsh sediments.

Composition of PAHs can indicate different contamination sources, including petroleum and low-medium vs. high temperature alteration of organic matter [13,60]. To evaluate contamination sources for the PAHs, we applied principal component analysis (PCA) using the compositional data [4,61-63]. Principal component 1 (PC1) and PC2 accounted for 61% and 20% variance of the data matrix, respectively (Figure 7). From the plot, shelf sediments were well separated from marsh sediments, which were enriched with PAHs with 4–6 rings, including BaA, Chr, BbkF, BaP, BahA and BghiP. These high-molecular-weight PAHs are typically from gasoline and diesel combustion [42,64-66]. In contrast, shelf sediments were enriched with low-molecular-weight PAHs, which are abundant in crude oil. These results suggest that petroleum combustion contributes more to the PAHs in marsh sediments, while uncombusted petroleum contributes more to the PAHs in shelf sediments.

Ratios of selected PAH isomers, including Phe/(An + Phe), Flua/(Flua + Pyr), Chr/(BaA + Chr) and InP/(InP + BgP), can also help to differentiate contamination sources [67,68]. Ratios of InP to 276 (InP and BgP) and Flua to 202 (Flua and Pyr) were applied to our data, because these isomers have great stability [69]. For most sediment samples except Sta. B1, both InP/276 and Flua/202 ratios were between 0.2 and 0.5 and below 0.5, respectively. This result suggests that PAHs in all the northern Gulf of Mexico sediments were from mixed sources of petroleum and fossil fuel combustion [70], even though combustion sources contribute more to marsh sediments and petroleum contributes more to shelf sediments.

In addition to contamination source, particle size may affect PAH composition [71]. Organic matter in finer particles is often more degraded than that in coarser particles [72,73], so PAHs may sorb differently to particles with different sizes. For example, high-molecular-weight PAHs were found to be more abundant in finer than coarser fractions after salt marsh sediments were size-fractionated [59]. This argument seems to be supported by the different PAH compositions between Sta. B1 and Sta. B2. Sta. B1 contained a higher proportion of 2-ring PAHs (>40%) and a lower proportion of 5-6-ring PAHs (<20%), while Sta. B2 contained a lower proportion of 2-ring PAHs (<20%) and more 5-6-ring PAHs (>35%) (Figure 7). As these two sites are only 3 km away from each other and thus expected to have similar contamination sources, this drastic compositional difference suggests that PAHs may have been redistributed within this area by strong physical dynamics, such as freshwater inflows and tidal currents. High-molecular-weight PAHs, which are more hydrophobic, may preferentially bind with finer mineral grains rich in degraded OM, and low-molecular-weight PAHs with coarser mineral grains with fresher OM. Such a pattern can be explained by the fact that degraded organic matter is more “glassy” [72,73], and has stronger sorption capability for PAHs with 5–6 rings compared to fresher organic matter in coarser size fractions [74-76].

### Bioavailable PAHs

A large fraction of PAHs is often strongly sorbed to sedimentary OM, so only a small fraction is bioavailabile. The fraction that can be digested by organisms may affect the functioning of coastal ecosystems, so quantifying the bioavailable PAHs is important for risk assessment and bioremediation [77,78]. The bioavailable PAHs here were quantified using a cocktail of sodium taurocholate and protein, as an analog of the digestive fluids in deposit-feeding macrofauna [38]. While these bioavailable percentages were very low, it is likely that repeated extractions would have released more PAH [38]. These extractabilities are lower than those in experiments with freshly spiked PAH in sediment [39], and are likely due to the decrease in extractability that often accompanies aging of PAH in sediment [79].

The percentages of bioavailable PAHs were negatively correlated (R = 0.88, p < 0.05) in sediments with high organic carbon contents, similar to the effect of sedimentary organic carbon concentration on gut fluid extractability of hydrophobic methyl mercury [80]. High contents of OM can compete more effectively with digestive fluids for PAHs, leading to smaller bioavailable fractions [81]. For example, Nam et al. (1998) [81] found that more PAHs are sequestered in sediments with more organic matter [82]. The bioavailability of PAHs may be also influenced by mineral surface area, particularly in sediments with low organic content [57,83,84]. Higher fractions of bioavailable PAHs were found in sediments with lower mineral surface area in this study, even though an inverse correlation was not significant (R = 0.71, p < 0.1). It is also clear that PAHs with low molecular weights had higher bioavailable percentages than those with high molecular weights (Figure 4). Lower bioavailability for PAHs with more benzene rings indicates the bioavailability of PAHs decreased with their octanol-water partition coefficient (K_ow_) [85]. Even though the bioavailable fractions only account for 0.05%, more bioavailable PAHs in marsh sediments indicates higher risk to organisms in marshes than those in the shelf sediments.

## Conclusion

In this study we provided baseline data of the PAH distribution in both surface marsh and shelf sediments of the northern Gulf of Mexico, identified contamination sources for the PAHs, and investigated factors controlling concentration and composition of PAHs. The PAH concentrations in the study area were below effects low-range, ranging from 100–856 ng g^-1^, indicating little toxicity to organisms. The levels of PAHs followed the trend of MRM > marsh > shelf sediments. PAHs concentrations were positively correlated with C/N ratios of OM, suggesting that PAHs preferentially bind with terrestrial organic matter. PAHs with 3–4 rings were dominant in all the sediments, but the PAH compositions differed between marsh and shelf sediments. The PCA results showed that PAHs in marsh sediments were primarily pyrogenic, while PAHs in shelf sediments were primarily petrogenic. Particle size also affected PAHs compositions, as shown via strong size-composition within small regions. The fraction of bioavailable PAHs was negatively correlated with specific mineral surface area or organic carbon contents in sediments, indicating that a stronger association of PAHs with fine particles decreases their bioavailability.

## Competing interests

The authors declare that they have no competing interests.

## Authors’ contributions

ZW carried out most of the analyses, interpreted the results and drafted the manuscript. ZL designed the experiments and helped interpret and draft the manuscript. KX analyzed the grain size distribution of the sediment. LMM analyzed sediment CHN contents and mineral surface area, and extracted the bioavailable PAHs. ZZ helped the PAH analysis. ASK and WW helped sample collection. All authors read and approved the final manuscript.
